# Homozygous missense variant in the *TTN* gene causing autosomal recessive limb-girdle muscular dystrophy type 10

**DOI:** 10.1186/s12881-019-0895-7

**Published:** 2019-10-29

**Authors:** Amjad Khan, Rongrong Wang, Shirui Han, Muhammad Umair, Safdar Abbas, Muhammad Ismail Khan, Mohammad A. Alshabeeb, Majid Alfadhel, Xue Zhang

**Affiliations:** 1 McKusick-Zhang Center for Genetic Medicine, State Key Laboratory of Medical Molecular Biology, Institute of Basic Medical Sciences Chinese Academy of Medical Sciences, School of Basic Medicine Peking Union Medical College, Beijing, China; 2 The Research Center for Medical Genomics, China Medical University, Shenyang, China; 3 Developmental Medicine Department, King Abdullah International Medical Research Center (KAIMRC), Ministry of National Guard-Health Affairs (MNGHA), King Saud Bin Abdulaziz University for Health Sciences, Riyadh, Saudi Arabia; 4 Department of Biochemistry, Faculty of Biological Sciences, Quaid-i-Azam University, Islamabad, Pakistan; 5 Medical Genomics Research Department, King Abdullah International Medical Research Center (KAIMRC), Ministry of National Guard-Health Affairs (MNGHA), King Saud Bin Abdulaziz University for Health Sciences, Riyadh, Saudi Arabia; 6 Department of Zoology, Islamia College University, Peshawar, Pakistan

**Keywords:** LGMD, Consanguineous family, TTN, Whole exome sequencing

## Abstract

**Background:**

Limb-girdle muscular dystrophies (LGMDs) are large group of heterogeneous genetic diseases, having a hallmark feature of muscle weakness. Pathogenic mutations in the gene encoding the giant skeletal muscle protein titin (TTN) are associated with several muscle disorders, including cardiomyopathy, recessive congenital myopathies and limb-girdle muscular dystrophy (LGMD) type10. The phenotypic spectrum of titinopathies is expanding, as next generation sequencing (NGS) technology makes screening of this large gene possible.

**Aim:**

This study aimed to identify the pathogenic variant in a consanguineous Pakistani family with autosomal recessive LGMD type 10.

**Methods:**

DNA from peripheral blood samples were obtained, whole exome sequencing (WES) was performed and several molecular and bioinformatics analysis were conducted to identify the pathogenic variant. *TTN* coding and near coding regions were further amplified using PCR and sequenced via Sanger sequencing.

**Results:**

Whole exome sequencing analysis revealed a novel homozygous missense variant (c.98807G > A; p.Arg32936His) in the *TTN* gene in the index patients. No heterozygous individuals in the family presented LGMD features. The variant p.Arg32936His leads to a substitution of the arginine amino acid at position 32,936 into histidine possibly causing LGMD type 10.

**Conclusion:**

We identified a homozygous missense variant in *TTN*, which likely explains LGMD type 10 in this family in line with similar previously reported data. Our study concludes that WES is a successful molecular diagnostic tool to identify pathogenic variants in large genes such as *TTN* in highly inbred population.

## Background

Limb-girdle muscular dystrophies (LGMDs) are clinically and genetically heterogeneous muscle disorders inherited as an autosomal recessive or dominant pattern. Clinically, patients are characterized by symmetrical weakness of pelvic, scapular and trunk muscles [1, 2]. LGMDs also show clinical overlapping with other muscle disorders like Emery-Dreifuss Muscular Dystrophy (EDMD; MIM: 310300), recessive congenital myopathy [MIM: 612540], myofibrillar myopathy (MFM; MIM: 601419) and late onset dominant distal myopathy [MIM: 604454] [3, 4]. More than 30 recessively and dominantly inherited forms have been identified for LGMDs [3]. The prevalence of LGMDs is about 4–7/100,000 and may have childhood, teenage or adulthood onset [3, 4]. The prevalence of autosomal recessive muscle disorders like LGMD and congenital muscular dystrophies are rare in Pakistani populations. LGMD shows severe clinical manifestations such as proximal muscle weakness, loss of ambulation between third and sixth decade, severe disability within 20 years of onset, and muscle biopsy might reveal dystrophic changes [3, 4]. Patient with LGMD had a similar disease course as Duchene muscular dystrophy (DMD), had calf hypertrophy and were non-ambulatory after age 15. Pathogenic mutations in *TTN* has also been associated with other severe disorders such as cardiomyopathy, dilated, 1G (MIM:604145), cardiomyopathy, familial hypertrophic 9 (MIM:613765), muscular dystrophy, limb-girdle, autosomal recessive 10 (MIM:608807), myopathy, proximal, with early respiratory muscle involvement (MIM:603689), salih myopathy (MIM:611705), tibial muscular dystrophy, tardive (MIM:600334) [5–10] .

In this study, we documented a clinical and molecular investigation of a consanguineous Pakistani family segregating LGMD in an autosomal recessive form and identified a novel homozygous missense mutation in the *TTN* gene located on chromosome 2q31.2. To the best of our knowledge the molecular studies on mutation in the *TTN* gene is reported for the first time from Pakistan.

## Methods

### Family recruitment and DNA isolation

**Fig. 1:**
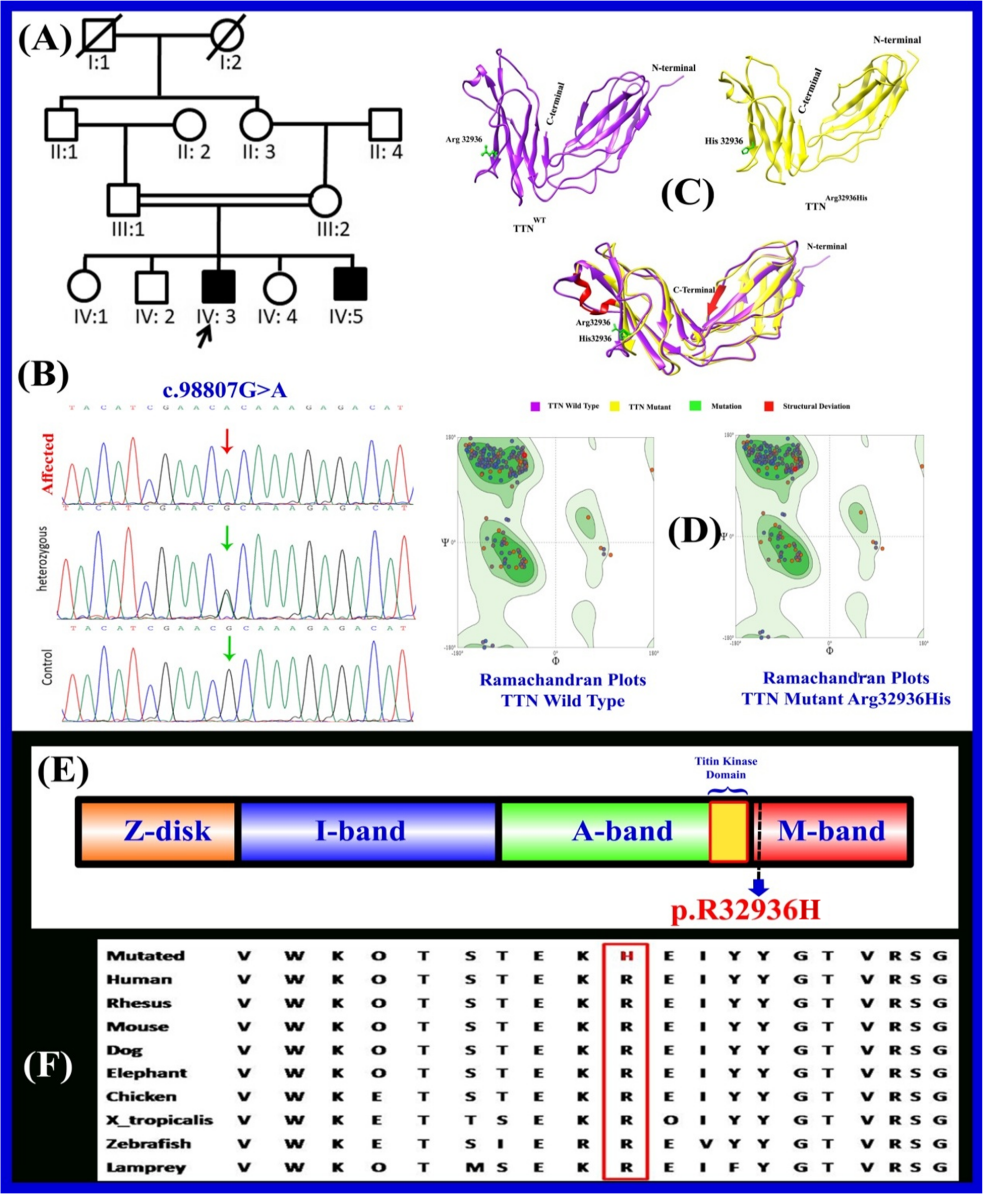
**a** A consanguineous pedigree showing two affected members (IV:3 and IV:5) in the fourth generation having limb girdle muscular dystrophy. Affected individuals in the pedigree are shown with shaded symbols and unaffected with open symbols. Double lines indicate consanguineous union. **b** Sequence chromatogram of the *TTN* gene is showing segregation of c.98807G > A; p. Arg32936His in all family members **c** Ribbon representation of three-dimensional structure of human titin with close-up view of mutant (right) and wild type (left) at position 32,936 showing the local conformation induced by the substitution of arginine by histidine. **d** Ramachandran plots of wild and mutant types. **e** Schematic view of the functional domain of the *TTN* gene and localization of known mutation (Arg32936His). The novel missense variant p. Arg32936His reported here is indicated in red localized in the M domain. **f** The panel also shows the evolutionary conservation of Arg32936 across different species

The present family has two affected individuals lives in the Bannu district of Khyber Pakhtunkhwa province, Pakistan. Pedigree was drawn (Fig. 1a) and the affected individuals were thoroughly examined by a local medical doctor. Clinical information including age, gender, family history and consanguinity was recorded. Blood samples were drawn from the two affected (IV: 3, IV: 5) and normal individuals of the family. Genomic DNA was extracted using the QIAquick DNA extraction kit (QIAamp, Qiagen, Valencia, CA, USA) and quantified using Nanodrop-2000 spectrophotometer (Thermo Scientific, Schaumburg, IL, USA).

### Library preparation and whole-exome sequencing

A 100 ng of genomic DNA were needed to amplify the targeted amplicon. Exome libraries of the DNA product were created using the Ion AmpliSeq™ Exome Panel [11–13]. Emulsion polymerase chain reaction (emPCR) was performed using a OneTouch 2 instrument with an Ion PI Template OT2 200 Kit V3.The Ion OneTouch ES enrichment system (Life Technologies, Carlsbad, USA) was used for ISP enrichment step. The manufacturer’s instructions of Life Technologies company were followed to prepare and load the Ion Proton I chip [11–13].

### Data processing

Sequencing data were aligned to the *Homo sapiens* hg 19 (GRCh37/hg19). Torrent Variant Caller software (version 4.4.3) was used to analyze the genotyping data and call the multi-allelic variations and indels. Post detection of variant was performed using wANNOVAR (http://wannovar.usc.edu/). An Integrative Genome Viewer (IGV, http://www.broadinstitute.org/igv/) was used to visualize sequencing data. Variant frequencies were obtained from various databases such as the 1000 Genomes Project, dbSNP142, Exome Aggregation Consortium (ExAC) and gnomAD (Additional file 1: Table S1).

### Bioinformatics analysis

Different prediction programs including Polyphen-2 (http://genetics.bwh.harvard.edu/pph2/), SIFT (http://sift.jcvi.org/), PROVEAN (http://provean.jcvi.org) and Mutation Taster (http://www.mutationtaster.org/) predicted this mutations to be probably damaging. Finally, for the interpretation of variants, American College of Medical Genetics and Genomics (ACMG) 2015 guidelines were used [14].

### Mutation confirmation

To validate the detected variant, specific fragments were PCR-amplified using site-specific primers using primer3 software (http://primer3.ut.ee) and analyzed by Sanger sequencing (Fig. 1b). The identified variant was analyzed in 200 ethnically matched control individuals (Fig. 1).

### Protein modeling

The primary sequence of TTN was retrieved in FASTA format through UniProtKB/SwissProt database (https://www.uniprot.org/uniprot/O95672). Retrieved sequence was used to predict the three-dimensional (3D) protein structure using I-TASSER server (https://zhanglab.ccmb.med.umich.edu/I-TASSER/). The three-dimensional model of mutated TTN protein (p.Arg32936His) was generated by MODELLER 9.17 (https://salilab.org/modeller/9.17/release.html). The recognition of errors in experimental and theoretical models of protein structures is a major problem in structural bioinformatics. Different evaluation tools were used for the assessment of protein structure. The model was further processed by RAMPAGE (http://mordred.bioc.cam.ac.uk/~rapper/rampage.php) ERRAT (https://servicesn.mbi.ucla.edu/ERRAT/) and Protein Structure Analysis (ProSA; https://prosa.services.came.sbg.ac.at/prosa.php). RAMPAGE generates Ramachandran plot for the assessment of models along with distribution of residues in favoured, allowed and outlier regions. ERRAT generated a plot indicating the confidence and overall quality of model. ProSA calculated an overall quality score of the predicted structure (Fig. 1c & d).

## Results

### Clinical description of patients

Clinical examination was performed for both affected individuals (IV: 3; IV: 5). They were born to first-cousin parents with a normal pregnancy and delivery. They were 20–25 years old, and had severe LGMD. Notable clinical findings include difficulty in rising from the floor, delay in motor milestones, and muscle weakness. They had mild microcephaly, intellectual disability (ID), generalized muscle hypertrophy and developmental delay (Table 1). Follow up clinical examination of the patients revealed cardiomyopathy, proximal and distal weakness, inability to stand, loss of ambulation, and both were confined to a wheelchair. They also had a triangular face, low set of ears, and clinodactyly in lower limb digits. In addition, both were suffering pelvic and shoulder girdle muscular dystrophy, muscular pain and also facial muscles weakness when doing a usual muscle exercise. Features such as skin, teeth, nails, eyes, reproductive and cardiac deformities were not observed in both of them. Their parents showed no abnormalities and were healthy.

**Table 1:** Clinical features of the affected individuals

Variable	Subject (IV:3)	Subject (IV:5)
Sex	Male	Male
Age	25	20
Microcephaly	+	+
Wheelchair	+	+
Scoliosis	+	+
Synophrys	+	+
Hearing impairment	–	–
Intellectual disability	+	+
Pelvic girdle weakness	+	+
Skeletal abnormalities	++	++
Difficulty in rising from the floor	++	++
Syncope attack	+	+
Scapular and trunk muscles weakness	+	+
Cardiac impairment	+	+
Muscle pain and stiffness	–	–
Seizures	–	–
Cancer	–	–
Narrow shoulder	+	+
Skin	Normal	Normal
Eye sight	Normal	Normal
Behavior	Nervous/forgetful	Nervous/forgetful
Pregnancy event	Normal	Normal

+, present; ++, severe phenotype; −, absent

### Whole exome sequencing

In the present study, clinical diagnosis was confirmed by genetic analysis. Of these, both patients (IV: 3; IV: 5) and their parents (III-1;III-2) were subjected to whole exome sequencing (WES) as described earlier using Ion Torrent platform [11–13]. WES results indicated a novel homozygous missense variant (c.98807G > A; p.Arg32936His) in *TTN* (MIM: 188840) gene responsible for LGMD phenotype (Table 2). Sanger sequencing perfectly confirmed segregation of the disease phenotype. The variation G-to-A transversion results into the substitution of arginine (R) to histadine residues (Arg32936His). This mutation is conserved across different species and can affect greatly the amino acid (aa) sequence located in the M domain of *TTN* gene that might change the protein features and also affect the splice site (Fig. 1 e& f). Different online bioinformatics tools were used to analyze the pathogenicity of the variant (Table 2).

**Table 2:** Homozygous variant on chromosome 2 from exome data of *TTN* family

Family	Individuals (IV:3 and IV:5)
Chr. Position (hg19)	chr2:179403855
Reference allele	G
Alternate allele	A
Gene	*TTN*
MIM	188,840
Gene Bank	NM_001267550.2
ExonicFunc.refgene	nonsynonymous SNV
cDNA Change	c.98807G > A
Amino Acid change	p.Arg32936His
1000G_ALL	0.00
ExAC_Freq	0.0001019
dbSNP	rs774296358
ClinVar_Status	–
SIFT Score &prediction	0.044/D
Polyphen2 score & prediction	0.99/PD
Mutation taster score &predict	0.99/D
FATHMM_score & prediction	0.7881/D
CADD score	24.3/D
ACMG Classification	PM2
Variant Status	Novel
Other Information’s	Homozygous

*SNV Single Nucleotide Variant, *D* Damaging, *PD* Probably Damaging,*PM2* Pathogenic Moderate 2

Using homology modelling, 3D models of wild type and mutated TTN protein (p.Arg32936His) were predicted and evaluated by online structure analysis tools as described above. Ramachandran plot generated by RAMPAGE indicated that approximately 93% of residues in the model lie in allowed regions of torsion angles. ERRAT and ProSA showing overall quality of model and quality score of the predicted structure (Fig. 1 c & d).

## Discussion

LGMD is an inherited genetic disorder characterized by limb and girdle weakness and transmitted in either an autosomal recessive or an autosomal dominant pattern [1, 2]. Several genes are associated with the LGMD phenotype and the next generation sequencing (NGS) technology can be the best choice for definitive diagnosis of LGMD [15, 16]. The affected individuals reported here, exhibit several phenotypes such as difficulty in rising from the floor, delay in motor milestones, and muscle weakness, mild microcephaly, intellectual disability, generalized muscle hypertrophy and developmental delay (Table 1). Such features were also reported previously [15, 16]. Cardiomyopathy also was observed in our patients [17]. Recently, Younus et al (2019) reported a nonsense mutation in the SGCD gene among Pakistani population having LGMD features that shows variability with features in comparison the cases reported here [18]. Through WES, we detected a homozygous missense mutation (c.98807G > A; p.Arg32936His) in the exon 353 of the *TTN* gene known to be associated with LGMD phenotypes.

The titin protein is organized into four structurally and functionally distinct regions that correlate with the muscle sarcomere [19–21]. These regions, located at the amino terminus to the carboxy terminus of the protein, include the Z-disk, I-band, A-band, and the M-line [21–23] (Fig. 1). Carriage of the mutation c.98807G > A which is very close to the M domain of the *TTN* gene, results in amino acid change of the Arg32936 residue into the His32936 and alter the secondary structure of the TTN protein causing protein instability. Using homology modelling; three-dimensional models of wild-type and the mutated *TTN* protein (p.Arg32936His) revealed a Z scores between 0.5–1.0, indicating no significant deviation from the scores determined for proteins of similar size. The entire *TTN* gene consists of 364 exons, located on chromosome 2q31, and transcribes an mRNA over 100 kb long that could hypothetically produce around 38,138 residues and 4200 kDa proteins [24].

*TTN* has multiple key roles in all striated muscle cells, well suited for its role as an architectural protein and provide specific attachment to a plethora of essential proteins [23]. A total of 346 *TTN* disease-causing mutations (259 missense/nonsense, 23 splicing, 13 small insertions, 47 small deletions, 1 small indels and 2 gross deletions) have been reported in the human gene mutation database (HGMD) with at least 10 different conditions, including isolated cardiomyopathies, purely skeletal muscle phenotypes and infantile diseases affecting both types of striated muscles (Table 3) [17, 18]. A majority of patients with *TTN* mutations have normal intelligence quotient (IQ), but our patients showed poor language development, mild microcephaly and developmental delay (Table 1).

**Table 3:** HGMD reported mutations in *TTN* gene associated with LGMD disorders

Gene Name	DNA Variation	Protein Variation	Mutation type	Reported phenotype
*TTN*	c.187G > A	p.A63T	Missense	Muscular dystrophy, limb-girdle
*TTN*	c.3100G > A	p.V1034 M	Missense	Muscular dystrophy
*TTN*	c.7961G > A	p.R2654K	Missense	Muscular dystrophy
*TTN*	c.22771A > T	p.K7591*	Nonsense	Muscular dystrophy
*TTN*	c.28730C > T	p.P9577L	Missense	Muscular dystrophy
*TTN*	c.46363C > T	p.R15455*	Nonsense	Muscle weakness
*TTN*	c.49243G > A	p.A16415T	Missense	Muscular dystrophy
*TTN*	c.63658G > A	p.A21220T	Missense	Muscle weakness
*TTN*	c.76850G > A	p.R25617Q	Missense	Muscle weakness
*TTN*	c.78320C > T	p.P26107L	Missense	Muscle weakness
*TTN*	c.87483G > C	p.W29161C	Missense	Muscle weakness
*TTN*	c.97332C > A	p.Y32444*	Nonsense	Muscular dystrophy
*TTN*	c.98456C > G	p.S32819*	Nonsense	Muscle weakness
*TTN*	c.99274C > T	p.Q33092*	Nonsense	Muscular dystrophy
*TTN*	c.100133A > C	p.H33378P	Missense	Tibial muscular dystrophy
*TTN*	c.100136 T > A	p.I33379N	Missense	Tibial muscular dystrophy
*TTN*	c.100163 T > C	p.L33388P	Missense	Tibial muscular dystrophy
*TTN*	c.100186C > T	p.Q33396*	Nonsense	Tibial muscular dystrophy
*TTN*	c.57871 + 2 T > G	–	Splice site	Muscular dystrophy
*TTN*	c.99673 + 1G > C	–	Splice site	Muscle weakness
*TTN*	c.6379_6380delTA	p.(Tyr2127Leufs*8)	Deletion	Muscular dystrophy
*TTN*	c.43733-4_43740del12	–	Deletion	Muscular dystrophy
*TTN*	c.59385delT	p.(Lys19796Argfs*24)	Deletion	Tibial muscular dystrophy
*TTN*	c.90401delC	p.(Pro30134Leufs*15)	Deletion	Tibial muscular dystrophy
*TTN*	c.93409delT	p.(Ser31137Leufs*4)	Deletion	Muscular dystrophy, limb girdle 2 J
*TTN*	c.98807G > A	p.Arg32936His	Missense	Muscular dystrophy, limb-girdle
*TTN*	c.99943delT	p.(Ser33315Glnfs*10)	Deletion	Tibial muscular dystrophy
*TTN*	c.100185delA	p.(Lys33395Asnfs*9)	Deletion	Tibial muscular dystrophy
*TTN*	c.32190dupT	–	Duplication	Muscular dystrophy, limb girdle 2 J
*TTN*	c.92854_92857dupACTG	–	Duplication	Tibial muscular dystrophy
*TTN*	c.100076_100086delins11	–	Indels	Tibial muscular dystrophy
*TTN*	c.1662 + 15_3101–3	–	Gross deletion	Muscular dystrophy
*TTN*	ex. 34–41	–	Gross deletion	Muscle weakness

Homozygous knockdown mice *(ttn*−/−) had a degeneration of both distal and proximal skeletal muscles by 2–3 weeks of age [25]. *ttn*−/−mice developed a rigid gait, kyphosis due to axial skeletal muscle association and normally does not survive long. Histological studies indicated that degeneration was specific to skeletal muscles with no other symptoms such as cardiomyopathy or impairment of the central or peripheral nervous system [25]. Both fore and hind limbs skeletal muscles had severe and progressive dystrophic phenotypes indicating that *TTN* plays a pivotal role in skeletal system development [25].

## Conclusion

This is the first report of *TTN* pathogenesis causing LGMD type 10 from Pakistani population. Failure for detection of c.98807G > A (p. Arg32936His) in 200 ethnically matched control individuals chromosomes outside of the family or in the public databases, designate that this homozygous missense mutation (is probably pathogenic and deleterious. However, further studies regarding LGMDs among large number of Pakistani population might lead to a deeper understanding, genetic mechanisms and future therapeutic interventions.

## Supplementary information

**Additional file 1: Table S1.** Filtering steps followed to search for the candidate variant.

## Data Availability

The datasets supporting the conclusions of this article are included within the article and its additional file.

## Abbreviations

ACMG: American College of Medical Genetics and Genomics
DMD: Duchene muscular dystrophy
emPCR: Emulsion polymerase chain reaction
ERB: Ethical Review Committee
ExAC: Exome Aggregation Consortium
HGMD: Human gene mutation database
ID: Intellectual disability
LGMDs: Limb-girdle muscular dystrophies
NGS: Next generation sequencing
TTN: Titin
WES: Whole exome sequencing
