# Correction to: Homozygous missense variant in the TTN gene causing autosomal recessive limb-girdle muscular dystrophy type 10

**DOI:** 10.1186/s12881-019-0929-1

**Published:** 2019-12-12

**Authors:** Amjad Khan, Rongrong Wang, Shirui Han, Muhammad Umair, Safdar Abbas, Muhammad Ismail Khan, Mohammad A. Alshabeeb, Majid Alfadheland, Xue Zhang

**Affiliations:** 1 McKusick-Zhang Center for Genetic Medicine, State Key Laboratory of Medical Molecular Biology, Institute of Basic Medical Sciences Chinese Academy of Medical Sciences, School of Basic Medicine Peking Union Medical College, Beijing, China; 2 The Research Center for Medical Genomics, China Medical University, Shenyang, China; 3 Developmental Medicine Department, King Abdullah International Medical Research Center (KAIMRC), Ministry of National Guard-Health Affairs (MNGHA), King Saud Bin Abdulaziz University for Health Sciences, Riyadh, Saudi Arabia; 4 Department of Biochemistry, Faculty of Biological Sciences, Quaid-i-Azam University, Islamabad, Pakistan; 5 Medical Genomics Research Department, King Abdullah International Medical Research Center (KAIMRC), Ministry of National Guard-Health Affairs (MNGHA), King Saud Bin Abdulaziz University for Health Sciences, Riyadh, Saudi Arabia; 6 Department of Zoology, Islamia College University, Peshawar, Pakistan

**Correction to: BMC Med Genet (2019) 20:166**

**https://doi.org/10.1186/s12881-019-0895-7**

Please be advised that following publication of the original article [1], the authors have identified the following errors with the scientific content:

Fig. 1 (please note that the corrected Fig. 1 is enclosed in this article):

**Fig. 1:**
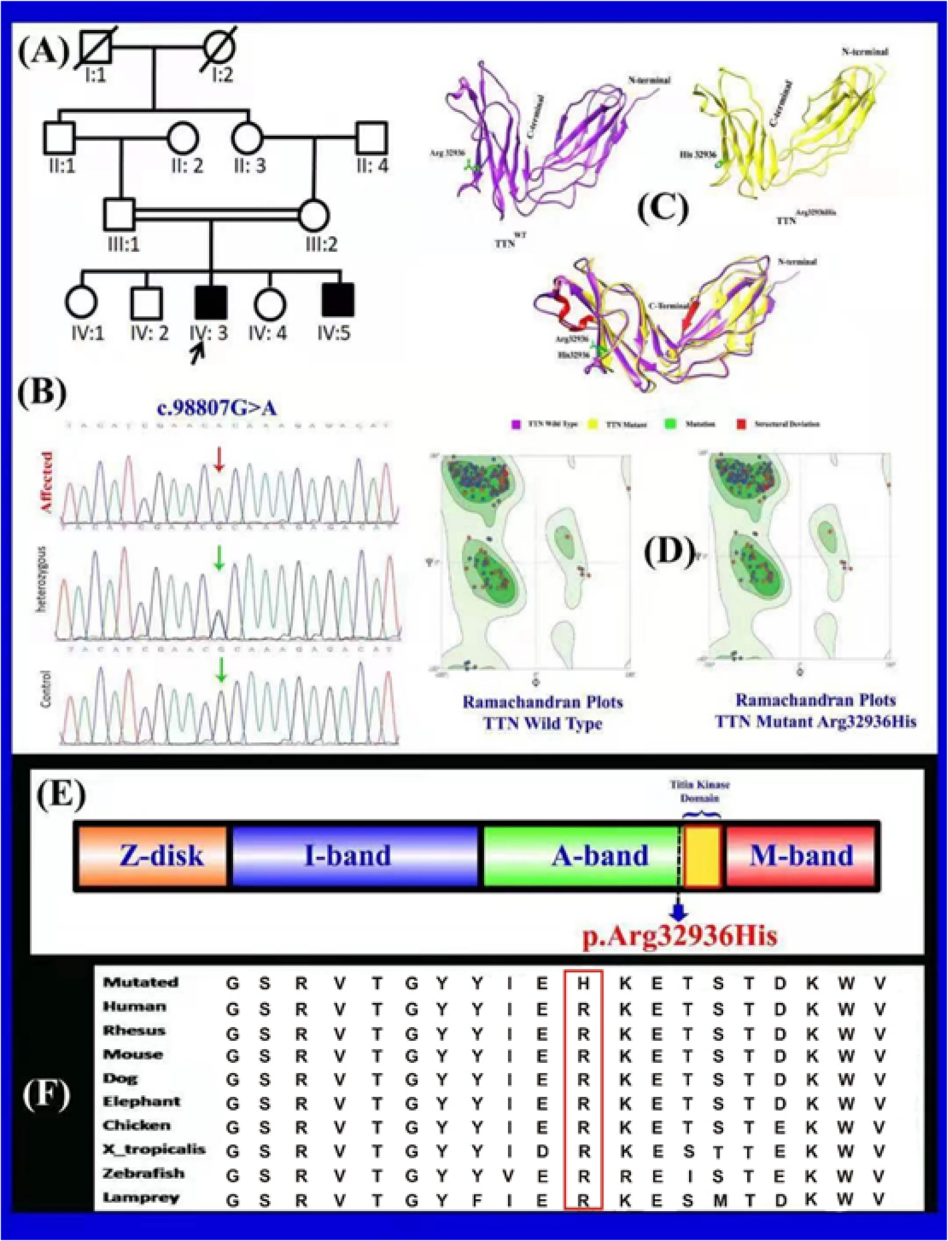
**a** A consanguineous pedigree showing two affected members (IV:3 and IV:5) in the fourth generation having limb girdle muscular dystrophy. Affected individuals in the pedigree are shown with shaded symbols and unaffected with open symbols. Double lines indicate consanguineous union. **b** Sequence chromatogram of the *TTN* gene is showing segregation of c.98807G > A; p. Arg32936His in all family members **c** Ribbon representation of three-dimensional structure of human titin with close-up view of mutant (right) and wild type (left) at position 32,936 showing the local conformation induced by the substitution of arginine by histidine. **d** Ramachandran plots of wild and mutant types. **e** Schematic view of the functional domain of the *TTN* gene and localization of known mutation (Arg32936His). The novel missense variant p. Arg32936His reported here is indicated in red localized in the FN3 domain 128, which is situated in the distal A-band region. **f** The panel also shows the evolutionary conservation of Arg32936 across different species

In part ‘(E)’, the arrow which indicates the location of the p. Arg32936His variant is incorrectly positioned

In part ‘(F)’, the amino acids should be in the N–C orientation, with the “O” letters replaced by “D”s

Legend of Fig. 1 (specifically, in the part concerning ‘1e’):

“(The novel missense variant p. Arg32936His reported here is indicated in red localized in the M domain)” should instead read “(The novel missense variant p. Arg32936His reported here is indicated in red localized in the FN3 domain 128, which is situated in the distal A-band region)”

In subsection (of the ‘Results’ section) ‘Whole genome sequencing’:

“the amino acid (aa) sequence located in the M domain of TTN gene” should instead read “the amino acid (aa) sequence located in the FN3 domain 128 of TTN gene”

In the ‘Discussion’ section:

“Carriage of the mutation c.98807G > A which is very close to the M domain of the TTN gene” should instead read “Carriage of the mutation c.98807G > A which is in the FN3 domain 128 of TTN gene”

The authors apologize for this error.
