# Supplementary material for: Homozygous missense variant in the TTN gene causing autosomal recessive limb-girdle muscular dystrophy type 10
Source: BMC Med Genet. 2019 Oct 29;20:166. doi: 10.1186/s12881-019-0895-7 (PMC6819411; doi:10.1186/s12881-019-0895-7)
Supplement: Supplementary file 1 — Additional file 1: Table S1. Filtering steps followed to search for the candidate variant. [file 12881_2019_895_MOESM1_ESM.docx]

**Supplementary Table 1 (S1):** Filtering steps followed to search for the candidate variant

| **Filtration methods** | **Number of Variants detected** |
| --- | --- |
| Total variants detected in affected individual (IV-3) | 76,773 |
| Total heterozygous variants detected | 43,934 |
| Total homozygous variants detected | 28,973 |
| Total variants after dbSNPs exclusion | 2,895 |
| Total homozygous frameshift variants detected | 140 |
| Total homozygous indels detected | 122 |
| Total homozygous missense variants detected | 70 |
| Total homozygous nonsense variants detected | 5 |
| Total homozygous splice site variants detected | 68 |
| Total homozygous near splice site variants detected | 18 |
| Total homozygous synonymous variants detected | 34 |
| Total homozygous unknown variants detected | 16 |
| Total homozygous 3’ and 5’ UTR variants detected | 310 |
| Total homozygous variants identified after applying different filters (NHLBI-ESP; 1000 Genomes; ExAC) with MAF>0.01 | 76 |
| Total compound heterozygous variants identified after applying different filters (NHLBI-ESP; 1000 Genomes; ExAC) with MAF>0.01 | 111 |
| Homozygous variant identified in limb girdle muscular dystrophy type 10 known gene and segregating with the disease phenotype in the family | 1 |
